# Diagnostic value of left atrial three-dimensional strain for paroxysmal atrial fibrillation in patients with acute ischemic stroke

**DOI:** 10.3389/fcvm.2026.1909840

**Published:** 2026-09-23

**Authors:** Yanni Wu, Xueyi Li, Chao Tang, Xiaorui Lv, Xia Zhang, Hui Li

**Affiliations:** 1 Department of Cardiology, The Second Affiliated Hospital of Soochow University, Suzhou, China; 2 Department of Neurology, The Second Affiliated Hospital of Soochow University, Suzhou, China

**Keywords:** acute ischemic stroke, paroxysmal atrial fibrillation, left atrial three-dimensional strain, echocardiography, diagnostic value

## Abstract

**Objective:**

To investigate the diagnostic and incremental value of left atrial three-dimensional (3D) strain parameters for paroxysmal atrial fibrillation (PAF) detected by short-term in-hospital cardiac monitoring and early Holter recording in patients with acute ischemic stroke (AIS).

**Methods:**

A total of 340 consecutive AIS patients were enrolled and divided into non-PAF (*n* = 282) and PAF (*n* = 58) groups. A base diagnostic model was constructed using univariable logistic regression and least absolute shrinkage and selection operator (LASSO) regression. The optimal 3D strain parameter was selected via receiver operating characteristic (ROC) curve analysis and added to the base model. Incremental value was assessed using C-index, net reclassification improvement (NRI), integrated discrimination improvement (IDI), and decision curve analysis (DCA). Model robustness was validated with bootstrap resampling.

**Results:**

All 3D left atrial strain parameters showed diagnostic value for PAF [all area under the curve (AUC) > 0.6]. The base model incorporating age, sex, B-type natriuretic peptide (BNP, log-transformed), and left atrial maximum volume index (LAVI) yielded a C-index of 0.910. After adding left atrial reservoir-phase circumferential strain (LASr-c), the C-index increased to 0.932, with a continuous NRI of 0.8638 and IDI of 0.0973. Bootstrap validation confirmed model robustness (bias-corrected C-index = 0.919). DCA showed that the new model provided greater net clinical benefit across most risk threshold ranges.

**Conclusion:**

Left atrial 3D strain has good diagnostic value for in-hospital detected PAF in AIS patients. Adding LASr-c may improve PAF risk stratification and inform subsequent targeted rhythm monitoring.

## 1 Introduction

Acute ischemic stroke (AIS) is one of the leading causes of adult disability and mortality worldwide, with cardioembolism representing an important pathogenic mechanism (1, 2). Paroxysmal atrial fibrillation (PAF) is the most common risk factor for cardioembolic stroke (3). However, its insidious onset and short duration make early clinical diagnosis challenging and result in a high missed diagnosis rate (4). When AIS is complicated by PAF, the risk of stroke recurrence is significantly higher than in patients without atrial fibrillation (AF) (5). Early identification of patients at high risk of PAF, followed by targeted extended rhythm monitoring and subsequent guideline-directed secondary prevention, can substantially reduce the risk of recurrent stroke (6). Therefore, developing a noninvasive, convenient, and generalizable approach for PAF risk stratification in patients with AIS, to inform clinical decisions regarding prolonged cardiac monitoring, is a major clinical imperative in cardiovascular and cerebrovascular medicine.

Left atrial (LA) structural and functional remodeling is the core pathophysiological basis for the initiation and maintenance of AF (7). Previous studies have mainly assessed LA remodeling using structural parameters such as LA diameter and LAVI. However, in PAF, LA functional impairment often precedes structural abnormalities, rendering conventional two-dimensional (2D) echocardiographic parameters inadequate for detecting early subclinical LA dysfunction (8, 9). Histological studies have demonstrated that LA strain parameters are significantly negatively correlated with the degree of atrial fibrosis and serve as reliable noninvasive surrogate markers (10). Furthermore, LA strain abnormalities are memory markers of atrial myocardial stress, inflammation, and metabolic disturbances; they can capture subclinical atrial remodeling before structural enlargement occurs, providing a solid pathophysiological rationale for early AF detection (11). Left atrial 3D strain technology enables real-time, full-angle assessment of myocardial deformation without angle dependency. It quantifies functional changes in the LA reservoir, conduit, and contractile phases with greater precision and offers higher sensitivity for identifying early LA functional impairment (12).

To date, accumulating evidence has confirmed the predictive value of 2D LA strain for occult PAF in patients with cryptogenic stroke. Two independent meta-analyses consistently demonstrated that new-onset AF in cryptogenic stroke is significantly associated with reduced left atrial reservoir and contractile strain as well as increased LAVI, with established cut-off values showing favorable discriminative ability (13, 14). What's more, a 2024 systematic review of 29 studies further characterized a gradient of LA structural and functional impairment across stroke subtypes, and the prospective ARIES study corroborated a stepwise decline in LA strain from normal sinus rhythm to high atrial ectopic burden and definitive AF in cryptogenic stroke populations (15, 16)\. However, nearly all of these data are derived from 2D strain assessments; research on the incremental diagnostic value of three-dimensional speckle-tracking echocardiography (3D-STE) in the general AIS population remains limited (17, 18). Existing 3D LA strain studies have primarily focused on AF risk prediction in the general population or assessment of recurrence after AF ablation (19, 20). Few studies have systematically evaluated the clinical utility of 3D LA strain for PAF screening in AIS patients and its incremental yield over conventional clinical and echocardiographic indicators.

Accordingly, this study aimed to systematically evaluate the performance of left atrial 3D strain parameters for PAF risk stratification in hospitalized patients with early-stage AIS, to construct a prediction model integrating conventional clinical and echocardiographic indicators, and to quantify the incremental value and clinical utility of adding 3D strain for guiding prolonged rhythm monitoring. By focusing on a general AIS cohort rather than only cryptogenic stroke, and using rigorous incremental value metrics, this study provides evidence-based support for noninvasive PAF screening in routine clinical practice.

## 2 Materials and methods

### 2.1 Study population

A total of 340 eligible patients with AIS admitted to the Department of Neurology of The Second Affiliated Hospital of Soochow University between January 2022 and December 2024 were consecutively enrolled (Figure 1).

**Figure 1:**
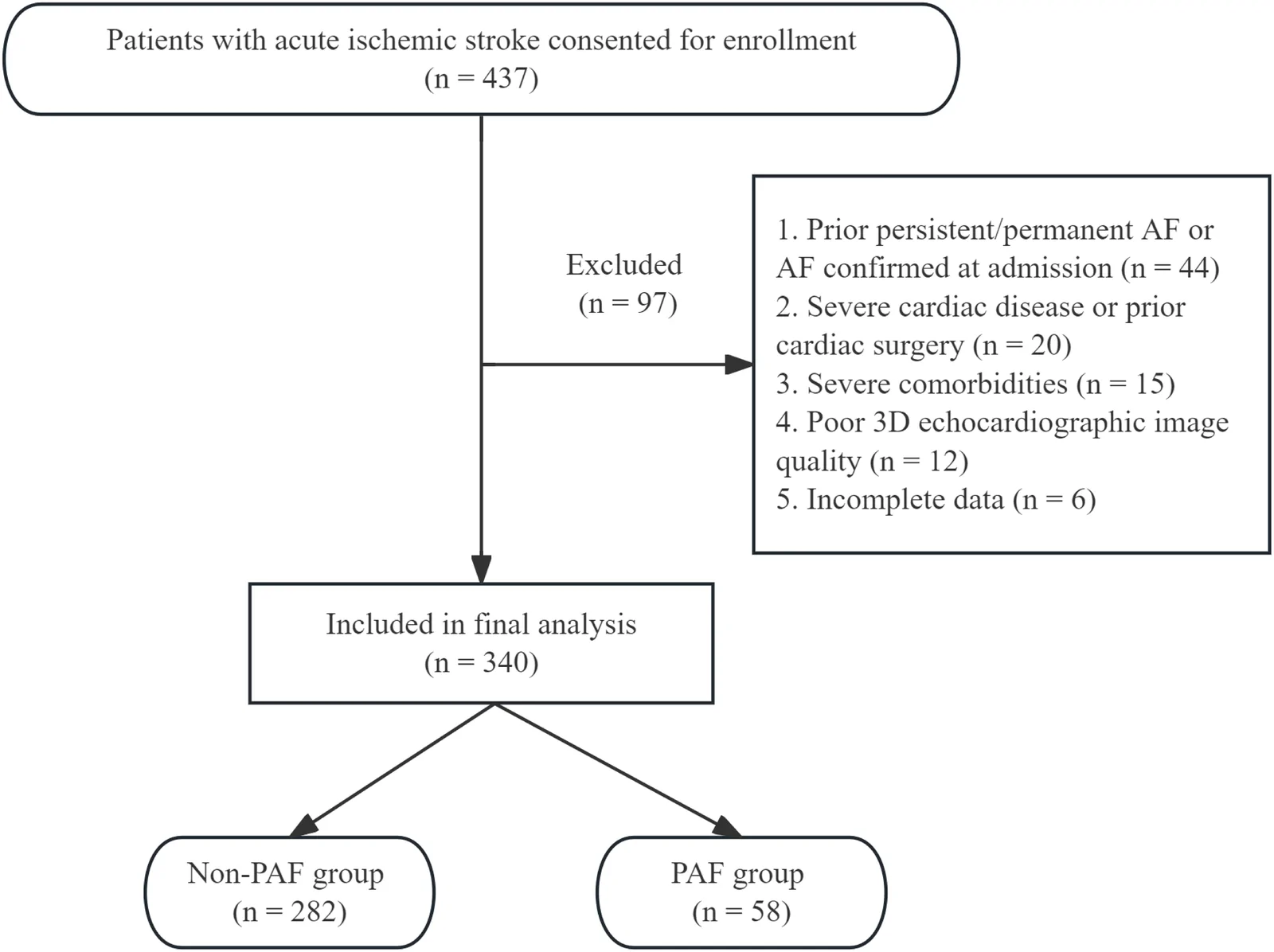
Flowchart detailing the patient selection process. AF, atrial fibrillation; PAF, paroxysmal atrial fibrillation.

Inclusion criteria: (1) Diagnosis of AIS according to standard clinical criteria and confirmed by cranial computed tomography (CT) or magnetic resonance imaging (MRI); (2) admission within 7 days of symptom onset; (3) complete transthoracic echocardiographic examination with left atrial 3D strain parameters measured after admission; (4) standardized electrocardiographic monitoring to ascertain the presence or absence of PAF.

Exclusion criteria: (1) history of persistent/permanent AF or AF documented on the admission 12-lead electrocardiogram (ECG); (2) severe valvular heart disease, dilated or hypertrophic cardiomyopathy, congenital heart disease, pericardial disease, or other cardiac conditions known to significantly affect LA structure and function; (3) previous cardiac surgery or radiofrequency catheter ablation; (4) end-stage diseases including severe hepatic or renal insufficiency, malignancy, or severe systemic infection; (5) suboptimal echocardiographic image quality precluding 3D strain analysis; (6) incomplete clinical data or refusal to participate.

### 2.2 Grouping

All patients underwent a stepwise inpatient electrocardiographic monitoring protocol for PAF detection. A 12-lead surface ECG was performed immediately on admission. Patients then received continuous inpatient cardiac monitoring (bedside ECG or telemetry) for at least 48 h; the total duration was determined by the attending physician based on the patient's clinical status, with no intervention from the study investigators. One 24-hour Holter monitoring test was started on the same day that continuous cardiac monitoring was discontinued.

PAF was diagnosed when any episode of atrial fibrillation lasting ≥30 s and terminating spontaneously within 7 days was recorded (21, 22). All electrocardiographic recordings were reviewed and adjudicated by trained clinicians. Patients were classified into the PAF group and non-PAF group according to the combined results of all above-mentioned electrocardiographic assessments.

### 2.3 Data collection

Baseline clinical characteristics: Demographic data (age, sex, body mass index), medical history (hypertension, diabetes mellitus, coronary heart disease, prior stroke, smoking and alcohol consumption), and medications on admission [angiotensin-converting enzyme inhibitors/angiotensin receptor blockers (ACEI/ARB), beta-blockers, antiplatelet agents such as aspirin and clopidogrel, and statins such as atorvastatin and rosuvastatin] were collected.

Stroke-related information: Time from symptom onset to admission, infarct location, infarct size, Trial of Org 10172 in Acute Stroke Treatment (TOAST) classification, Oxfordshire Community Stroke Project (OCSP) classification, and admission National Institutes of Health Stroke Scale (NIHSS) score were recorded.

Laboratory parameters: Fasting venous blood samples were obtained on the morning after admission. Complete blood count, coagulation profile, myocardial injury markers (creatine kinase-MB, myoglobin, troponin I), B-type natriuretic peptide (BNP), inflammatory markers (C-reactive protein), hepatic and renal function, fasting blood glucose, and lipid profile were measured.

Echocardiography: All examinations were performed by specialized echocardiographers blinded to the patients' clinical status using a standardized protocol. A GE VIVID E95 ultrasound system with a 4Vc probe was used to acquire dynamic images over five consecutive cardiac cycles. Patients were examined in the left lateral decubitus position during quiet respiration, with simultaneous ECG recording (to confirm sinus rhythm at the time of the examination). Conventional measurements included LA diameter, left ventricular end-diastolic diameter, left ventricular ejection fraction (LVEF), interventricular septal thickness, left ventricular posterior wall thickness, E/e' ratio, and LAVI. The acquired dynamic images were imported into a GE EchoPAC BT112 workstation, and the “4D Auto LAQ” mode was activated. The images were automatically rotated and cropped to generate three standard apical views: two-chamber, four-chamber, and three-chamber views. Left atrial views with clear endocardial borders were selected, and the left atrial structure was centered in each view. After identifying the central point of the mitral annulus, the software automatically traced the left atrial endocardial surface, with manual adjustment for tracing errors. The software then automatically calculated the left atrial longitudinal and circumferential strain values during the reservoir, conduit, and contractile phases (Figure 2). Intra-observer and inter-observer reproducibility were assessed in 10% of randomly selected patients. Intraclass correlation coefficients (ICC) and Bland-Altman plots demonstrated good reproducibility of the measurements (Supplementary Figure 1).

**Figure 2:**
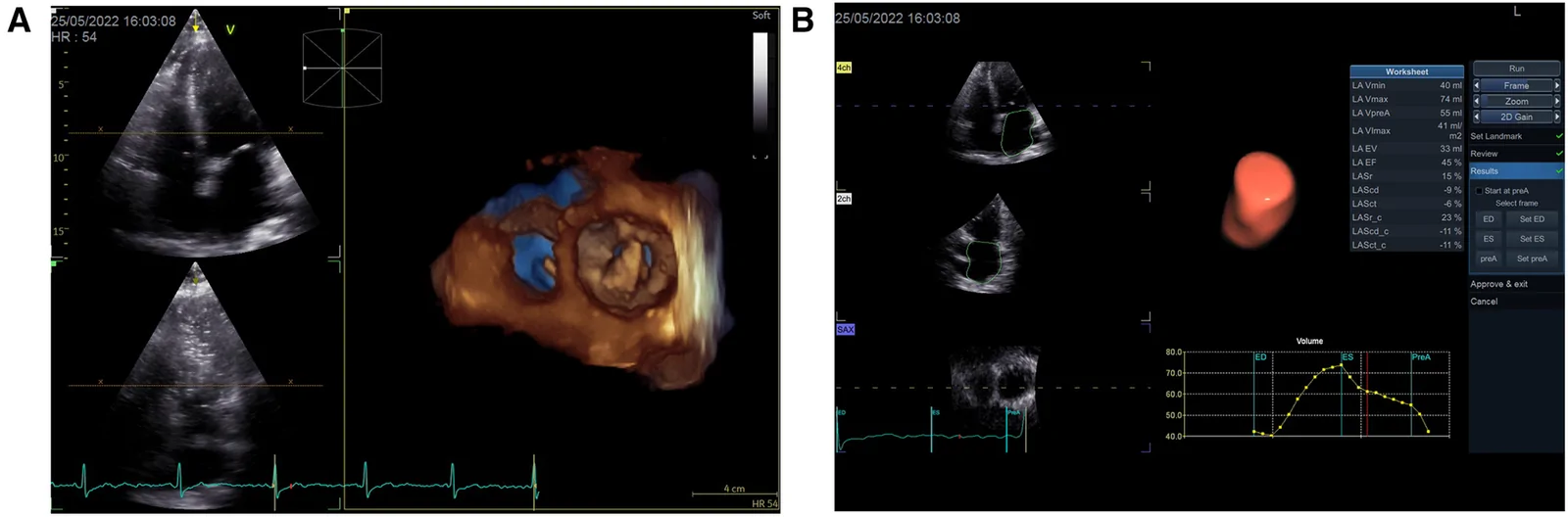
Three-dimensional left atrial imaging and strain analysis. **(A)** Three-dimensional full-volume imaging of the left atrium; **(B)** results of the analysis visualizing the left atrium 3D model and corresponding measurements in the right upper corner. The atrial cardiac cycle graph with corresponding volume tracing is in the right lower corner.

### 2.4 Statistical analysis

Statistical analyses were performed using R version 4.3.0 and MSTATA software (https://www.mstata.com/). The primary R packages employed included PredictABEL, pROC, rms, irr and rmda. The normality of all continuous variables was assessed using the Shapiro–Wilk test. Normally distributed continuous variables are presented as mean ± standard deviation and were compared using the independent-samples t-test; non-normally distributed continuous variables are presented as median (interquartile range) and were compared using the Mann–Whitney U test. Categorical variables are expressed as frequency (percentage) and were compared using the chi-square test or Fisher's exact test, as appropriate.

Univariable binary logistic regression was used to examine the association between each clinical indicator and PAF in AIS patients, with *P* < 0.05 as the threshold for initial screening. Variables identified as significant in the univariable analysis, together with the forced-in variables age and sex, were entered into a LASSO regression with 10-fold cross-validation repeated 100 times to select core predictors and construct the base diagnostic model. Receiver operating characteristic (ROC) curve analysis was performed to evaluate the diagnostic performance of each left atrial three-dimensional strain parameter; the area under the curve (AUC) with 95% confidence interval (CI) was calculated. The final selection of the optimal parameters for model augmentation was a *post-hoc* decision based on both physiological rationale and AUC value, and was not prespecified as the primary strain marker in the study protocol.

The overall discriminative ability of the base model versus the new model incorporating left atrial strain parameters was compared using the C-index and the DeLong test. Incremental diagnostic value was assessed using the continuous net reclassification improvement (NRI) and the integrated discrimination improvement (IDI). Model robustness was validated with 1 000 bootstrap resamples, and the bias-corrected C-index and optimism-corrected sensitivity/specificity were reported. Calibration curves for the base model and the new model were constructed, and the calibration slope, intercept, and Brier score of each model were further derived. Decision curve analysis (DCA) was used to evaluate the net clinical benefit of the diagnostic models.

To further verify the reliability of our findings, sensitivity analyses were conducted. First, patients with the CE-TOAST stroke subtype were excluded to validate the incremental diagnostic value of the optimal left atrial three-dimensional strain parameter for PAF in other stroke subtypes. Second, additional covariates were incorporated into the base model to further confirm the incremental value of the optimal left atrial three-dimensional strain parameter.

A two-sided *P* value < 0.05 was considered statistically significant.

## 3 Results

### 3.1 Baseline characteristics

A total of 340 AIS patients were enrolled, comprising 58 (17.1%) in the PAF group and 282 (82.9%) in the non-PAF group. Baseline characteristics of the two groups are presented in Table 1. Compared with the non-PAF group, patients with PAF were significantly older; had higher proportions of coronary heart disease and beta-blocker use; a shorter time from symptom onset to admission; higher prevalence of cortical infarction and cardioembolic stroke subtype; and significantly elevated levels of D-dimer, troponin I, BNP, C-reactive protein, serum creatinine, LA diameter, left ventricular end-diastolic diameter, and LAVI. In addition, total cholesterol, low-density lipoprotein cholesterol, LVEF, and the absolute magnitudes of all left atrial 3D strain parameters across the reservoir, conduit, and contractile phases were significantly lower in the PAF group (all *P* < 0.05). No significant differences were observed in sex, body mass index, hypertension, diabetes mellitus, prior stroke, infarct size, OCSP classification, complete blood count, or fasting blood glucose between the two groups (all *P* > 0.05).

**Table 1:** Baseline characteristics of the study population.

Characteristics	All (*n* = 340)	Non-PAF (*n* = 282)	PAF (*n* = 58)	*P* value
Baseline Demographics and Clinical Characteristics				
Age, years	67 (59, 75)	67 (58, 74)	73 (66, 82)	<0.001
Sex, male, *n* (%)	232 (68.2%)	197 (69.9%)	35 (60.3%)	0.156
BMI, kg/m²	23.9 (22.0, 26.0)	23.7 (21.8, 26.0)	24.1 (22.6, 26.1)	0.254
Smoking, *n* (%)	112 (32.9%)	99 (35.1%)	13 (22.4%)	0.061
Alcohol consumption, *n* (%)	51 (15.0%)	46 (16.3%)	5 (8.6%)	0.135
Hypertension, *n* (%)	238 (70.0%)	193 (68.4%)	45 (77.6%)	0.166
Diabetes mellitus, *n* (%)	107 (31.5%)	93 (33.0%)	14 (24.1%)	0.187
Coronary heart disease, *n* (%)	22 (6.5%)	14 (5.0%)	8 (13.8%)	0.034
Prior stroke, *n* (%)	69 (20.3%)	55 (19.5%)	14 (24.1%)	0.424
Antiplatelet agents, *n* (%)	84 (24.7%)	65 (23.0%)	19 (32.8%)	0.118
Statin, *n* (%)	99 (29.1%)	79 (28.0%)	20 (34.5%)	0.323
ACEI/ARB, *n* (%)	61 (17.9%)	49 (17.4%)	12 (20.7%)	0.549
*β*-blockers, *n* (%)	32 (9.4%)	21 (7.4%)	11 (19.0%)	0.006
Stroke-Related Characteristics				
Time from symptom onset to admission, hours	16 (4, 48)	20 (5, 52)	10 (3, 24)	0.021
Time from admission to Holter, days	6.0 (4.0, 8.0)	6.0 (4.0, 8.0)	6.0 (4.0, 8.0)	0.843
Infarct location, *n* (%)				0.002
Cortical	88 (25.9%)	63 (22.3%)	25 (43.1%)	
Deep	181 (53.2%)	162 (57.4%)	19 (32.8%)	
Multiple	39 (11.5%)	33 (11.7%)	6 (10.3%)	
Negative*	32 (9.4%)	24 (8.5%)	8 (13.8%)	
Infarct size, cm2	1.00 (1.00, 2.00)	1.00 (1.00, 2.00)	1.00 (1.00, 2.00)	0.500
TOAST classification, *n* (%)				<0.001
LAA	164 (48.2%)	147 (52.1%)	17 (29.3%)	
CE	44 (12.9%)	13 (4.6%)	31 (53.4%)	
SAA	103 (30.3%)	94 (33.3%)	9 (15.5%)	
SOE	15 (4.4%)	15 (5.3%)	0 (0.0%)	
SUE	14 (4.1%)	13 (4.6%)	1 (1.7%)	
OCSP classification, *n* (%)				0.439
TACI	27 (7.9%)	21 (7.4%)	6 (10.3%)	
PACI	105 (30.9%)	84 (29.8%)	21 (36.2%)	
POCI	89 (26.2%)	78 (27.7%)	11 (19.0%)	
LACI	119 (35.0%)	99 (35.1%)	20 (34.5%)	
Admission NIHSS score	2.0 (1.0, 4.0)	2.0 (1.0, 4.0)	2.0 (1.0, 4.0)	0.075
Laboratory Parameters				
White blood cell count, × 10⁹/L	6.80 (5.70, 8.10)	6.80 (5.60, 7.90)	7.25 (5.90, 8.50)	0.116
Red blood cell count, × 10¹²/L	4.57 (4.19, 4.91)	4.58 (4.21, 4.91)	4.42 (4.07, 4.83)	0.197
Hemoglobin, g/L	137 (126, 149)	138 (127, 149)	136 (121, 146)	0.160
Platelet count, × 10⁹/L	214 (177, 254)	215 (178, 252)	212 (168, 258)	0.717
Fibrinogen, g/L	2.97 (2.62, 3.47)	2.95 (2.61, 3.40)	3.18 (2.62, 3.67)	0.062
D-dimer, mg/L	0.36 (0.23, 0.59)	0.34 (0.22, 0.53)	0.48 (0.32, 0.72)	<0.001
CK-MB, U/L	2.1 (1.4, 5.9)	2.2 (1.4, 6.3)	2.1 (1.2, 3.3)	0.581
Myoglobin, ng/mL	39 (26, 100)	39 (25, 104)	43 (28, 77)	0.775
Troponin I, ng/L	11 (7, 16)	11 (7, 16)	15 (9, 21)	0.002
BNP, pg/mL	117 (58, 244)	102 (51, 179)	449 (214, 1,000)	<0.001
CRP, mg/L	6 (5, 6)	6 (5, 6)	6 (5, 7)	0.042
Serum creatinine, μmol/L	69 (60, 81)	68 (59, 79)	73 (64, 84)	0.040
Glucose, mmol/L	5.90 (5.23, 7.20)	5.81 (5.22, 7.20)	6.10 (5.24, 7.00)	0.882
Total cholesterol, mmol/L	4.35 (3.60, 5.03)	4.38 (3.67, 5.11)	4.09 (3.17, 4.85)	0.016
Triglycerides, mmol/L	1.38 (1.01, 1.91)	1.42 (1.03, 1.92)	1.24 (0.85, 1.87)	0.139
HDL-C, mmol/L	1.06 (0.90, 1.25)	1.05 (0.90, 1.25)	1.06 (0.91, 1.25)	0.936
LDL-C, mmol/L	2.72 (2.09, 3.27)	2.79 (2.15, 3.27)	2.37 (1.69, 3.26)	0.044
Homocysteine, μmol/L	13 (10, 16)	13 (10, 16)	14 (11, 16)	0.472
HbA1c, %	5.92 (5.60, 7.08)	5.90 (5.50, 7.12)	6.10 (5.70, 6.70)	0.512
Echocardiographic Parameters				
Time from symptom onset to LA strain measurement, days	6.0 (4.0, 8.0)	6.0 (4.0, 8.0)	6.0 (4.0, 8.0)	0.736
Heart rate, beat per minute	69 (62, 76)	69 (62, 76)	72 (64, 80)	0.172
LAD, mm	42.0 (39.7, 44.5)	41.5 (39.3, 43.9)	44.9 (41.5, 50.0)	<0.001
LVEDD, mm	47.5 (45.0, 50.0)	47.0 (44.7, 49.7)	49.5 (46.0, 53.0)	0.001
LVEF, %	66.1 (63.0, 69.9)	66.7 (63.3, 70.0)	65.0 (61.5, 67.3)	0.007
IVST, mm	9.90 (9.00, 10.39)	9.90 (9.00, 10.30)	10.00 (9.00, 10.40)	0.924
LVPWT, mm	9.60 (9.00, 10.00)	9.60 (9.00, 10.00)	9.63 (9.00, 10.00)	0.996
E/e' ratio	8.35 (7.00, 10.30)	8.29 (7.00, 10.11)	8.69 (7.23, 10.73)	0.239
LAVI, mL/m²	31 (25, 37)	29 (24, 34)	44 (35, 48)	<0.001
LASr, %	18 (14, 23)	20 (15, 24)	12 (7, 16)	<0.001
LAScd, %	−10.0 (−13.0, −6.0)	−10.0 (−13.0, −7.0)	−7.0 (−11.0, −4.0)	0.003
LASct, %	−9.0 (−13.0, −5.0)	−10.0 (−13.0, −6.0)	−3.5 (−7.0, 1.0)	<0.001
LASr-c, %	27 (20, 34)	28 (23, 35)	13 (6, 20)	<0.001
LAScd-c, %	−11 (−16, −7)	−12 (−17, −8)	−8 (−14, −4)	0.002
LASct-c, %	−15 (−21, −9)	−17 (−22, −12)	−4 (−10, 0)	<0.001

Continuous variables are presented as median (interquartile range) and categorical variables are presented as number of patients (%). *Imaging-negative stroke (no acute hyperintensity on DWI, with clinical symptoms and signs consistent with acute ischemic stroke). BMI, body mass index; ACEI, angiotensin-converting enzyme inhibitor; ARB, angiotensin receptor blocker; TOAST, Trial of Org 10172 in Acute Stroke Treatment; LAA, large-artery atherosclerosis; CE, cardioembolism; SAA, small-artery occlusion; SOE, stroke of other determined etiology; SUE, stroke of undetermined etiology; OCSP, Oxfordshire Community Stroke Project; TACI, total anterior circulation infarct; PACI, partial anterior circulation infarct; POCI, posterior circulation infarct; LACI, lacunar infarct; NIHSS, National Institutes of Health Stroke Scale; CK-MB, creatine kinase-MB; BNP, B-type natriuretic peptide; CRP, C-reactive protein; HDL-C, high-density lipoprotein cholesterol; LDL-C, low-density lipoprotein cholesterol; HbA1c, hemoglobin A1c; LA, left atrial; LAD, left atrial diameter; LVEDD, left ventricular end-diastolic diameter; LVEF, left ventricular ejection fraction; IVST, interventricular septal thickness; LVPWT, left ventricular posterior wall thickness; LAVI, left atrial maximum volume index; LASr, left atrial longitudinal strain reservoir phase; LAScd, left atrial longitudinal strain conduit phase; LASct, left atrial longitudinal strain contraction phase; LASr-c, left atrial circumferential strain reservoir phase; LAScd-c, left atrial circumferential strain conduit phase; LASct-c, left atrial circumferential strain contraction phase.

### 3.2 ROC analysis of left atrial three-dimensional strain parameters

As shown in Figure 3, all left atrial 3D strain parameters demonstrated discriminatory value for PAF in AIS patients (all AUC > 0.6). The highest AUC was obtained for LA circumferential strain contraction phase (LASct-c; AUC = 0.851, 95% CI: 0.797–0.906), followed by LA circumferential strain reservoir phase (LASr-c; AUC = 0.845, 95% CI: 0.780–0.911). No significant difference was found between the two parameters (*P* > 0.05), and both performed significantly better than all longitudinal strain parameters (all *P* < 0.05). Given their comparable discriminatory performance (*P* > 0.05 for difference in AUC), LASr-c was chosen for subsequent model integration as a *post-hoc* selection. The rationale was twofold: first, reservoir-phase circumferential strain directly reflects LA diastolic reserve function, an early marker of atrial fibrotic remodeling with clearer pathophysiological interpretability; second, contractile-phase strain is more susceptible to confounding by heart rate and loading conditions, whereas reservoir-phase strain has better measurement stability and is more suitable for clinical translation. Detailed diagnostic performance of all parameters is available in Supplementary Table 1. For all parameters, reduced absolute strain magnitude was associated with higher PAF risk.

**Figure 3:**
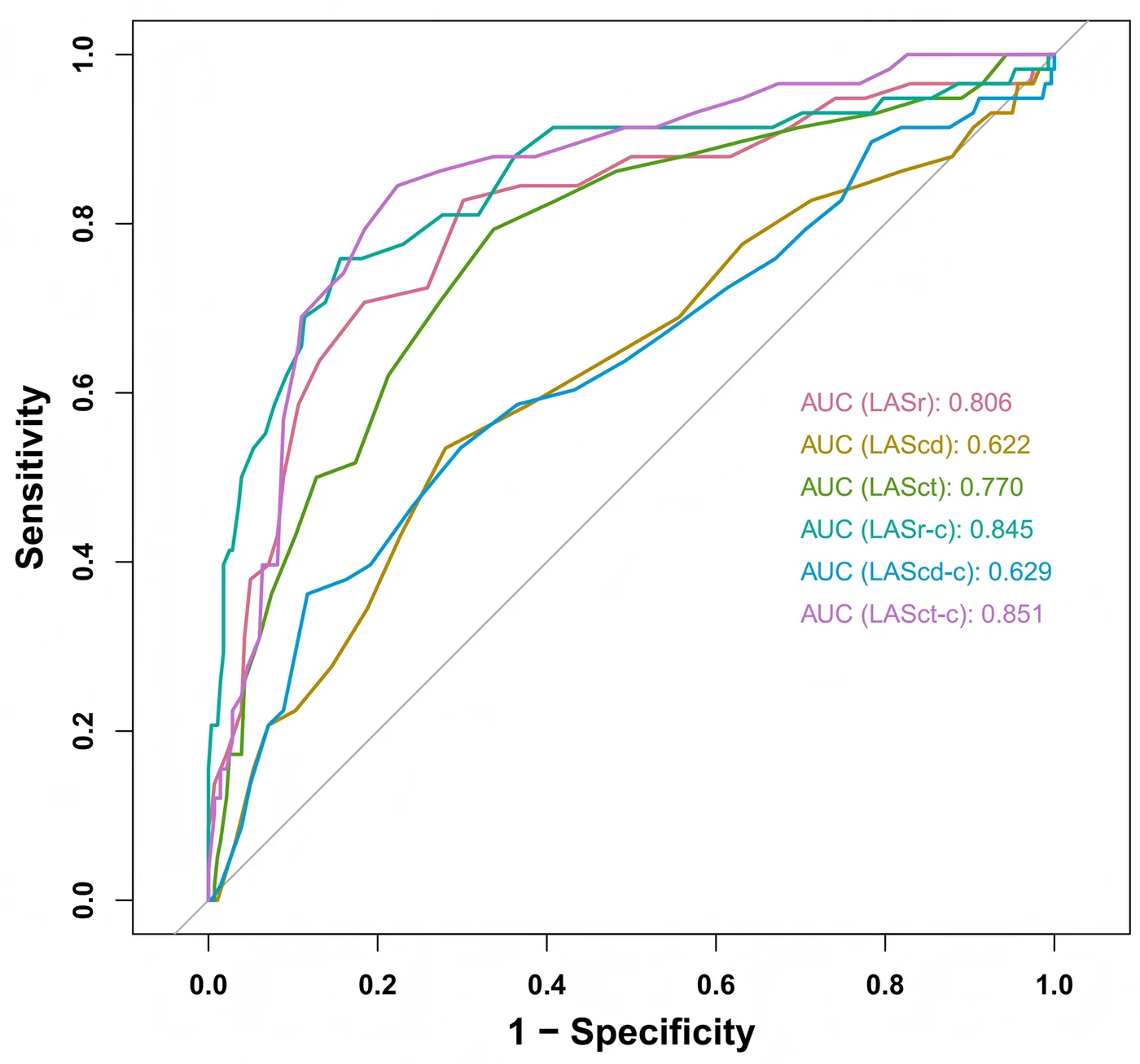
ROC curve analysis of three-dimensional LA strain parameters for detecting PAF in patients with AIS. LA. left atrial; LASr, LA longitudinal strain reservoir phase; LAScd, LA longitudinal strain conduit phase; LASct, LA longitudinal strain contraction phase; LASr-c, LA circumferential strain reservoir phase; LAScd-c, LA circumferential strain conduit phase; LASct-c, LA circumferential strain contraction phase.

### 3.3 Construction of the base diagnostic model

Univariable logistic regression was conducted for all baseline variables except left atrial strain parameters (Supplementary Table 2). Variables with *P* < 0.05 included age, coronary heart disease, beta-blocker use, time from onset to admission, infarct location, TOAST classification, troponin I, BNP, total cholesterol, LA diameter, left ventricular end-diastolic diameter, LVEF, and LAVI. After excluding TOAST classification to avoid circular reasoning with PAF diagnosis, and with forced inclusion of sex, a total of 13 variables entered LASSO regression (Figure 4). Using 10-fold cross-validation repeated 100 times and selecting *λ*.1se as the optimal penalty coefficient, two core predictors—BNP and LAVI—were retained. Together with the forced-in variables age and sex, a base diagnostic model with four indicators was constructed (For model interpretability, BNP was log-transformed and logBNP was entered into the final model; detailed information on the model is provided in Supplementary Table 3 and its footnotes).

**Figure 4:**
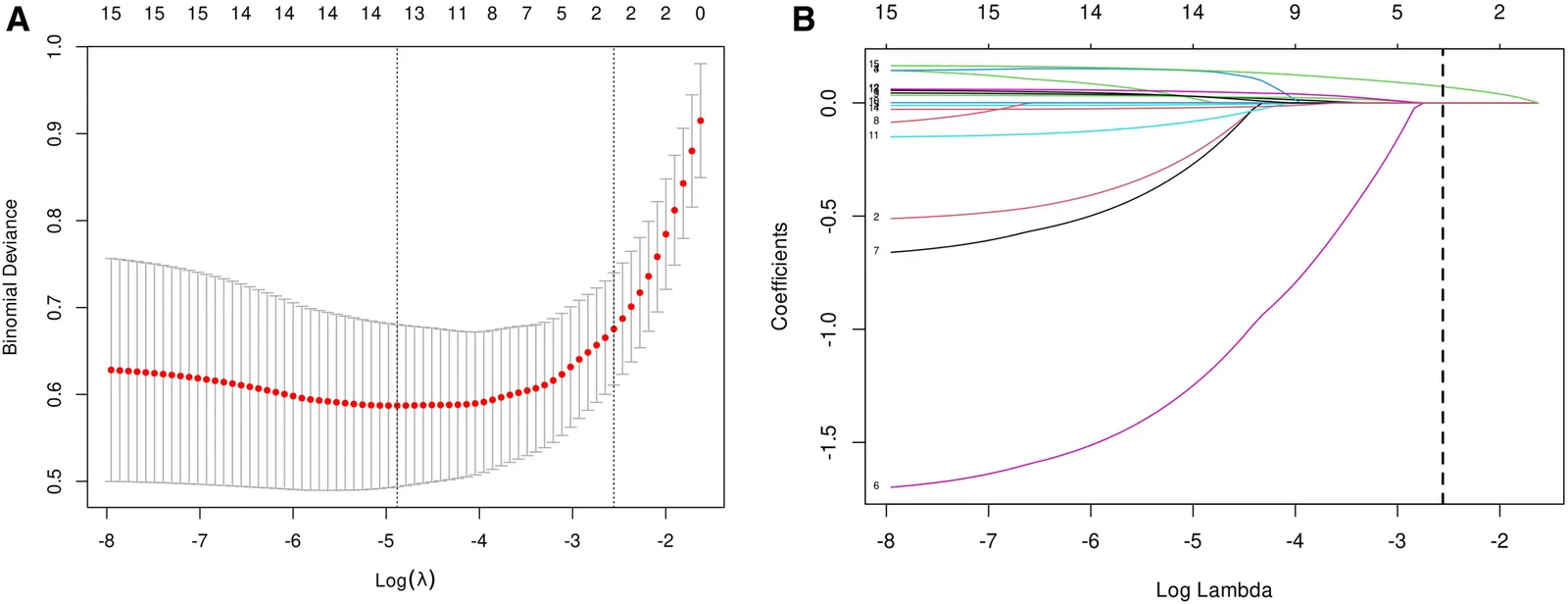
Variable selection using LASSO regression model. **(A)** Selection of the optimal penalty parameter (*λ*) via 10-fold cross-validation. The optimal *λ* value (*λ*.1se) was 0.0775; **(B)** LASSO regression coefficient profiles for all candidate variables. Variable number key: 1 = age, 2 = sex, 3 = coronary heart disease, 4 = beta-blocker use, 5 = time from onset to admission, 6 = infarct location (Deep), 7 = infarct location (Multiple), 8 = infarct location (Negative), 9 = troponin I, 10 = BNP, 11 = total cholesterol, 12 = LA diameter, 13 = left ventricular end-diastolic diameter, 14 = LVEF, 15 = LAVI.

### 3.4 Incremental value of LASr-cover the base model

The base model achieved a C-index of 0.910 (95% CI: 0.867–0.953). After adding LASr-c, the C-index of the new model improved to 0.932 (95% CI: 0.893–0.971); the DeLong test showed no statistically significant difference in AUCs (*P* = 0.199). However, reclassification and discrimination improvement analyses demonstrated that both continuous NRI and IDI were greater than zero with *P* < 0.05, indicating that adding LASr-c significantly improved risk reclassification and discriminatory ability for PAF (Table 2). Furthermore, the new model yielded higher sensitivity and specificity compared with the base model.

**Table 2:** Incremental diagnostic value of LASr-c over the base diagnostic model.

	Primary		Bootstrap 1,000 times	
	Base model	Base Model + LASr-c	Base model	Base Model + LASr-c
C-index (95% CI)	0.910 (0.867–0.953)	0.932 (0.893–0.971)	0.900 (0.844–0.942)	0.919 (0.874–0.952)
*P* value		0.199		
Cutoff value	0.153	0.178	-	-
Sensitivity, % (95% CI)	0.879 (0.793–0.948)	0.914 (0.828–0.983)	0.852 (0.661–0.947)	0.885 (0.661–0.967)
Specificity, % (95% CI)	0.823 (0.780–0.865)	0.887 (0.848–0.922)	0.819 (0.716–0.895)	0.884 (0.811–0.936)
Continuous NRI (95% CI)	Ref	0.864 (0.622–1.105)		
*P* value		<0.001		
IDI (95% CI)	Ref	0.097 (0.047–0.147)		
*P* value		<0.001		

LASr-c, left atrial circumferential strain reservoir phase; CI, confidence interval; NRI, net reclassification improvement; IDI, integrated discrimination improvement; Ref, reference.

### 3.5 Model robustness and net clinical benefit

Bootstrap validation with 1 000 resamples showed that the bias-corrected C-index of the new model incorporating LASr-c was 0.919 (95% CI: 0.874–0.952), closely matching the original C-index and indicating good model robustness. This value was still higher than the bias-corrected C-index of the base model, and the new model yielded higher optimism-corrected sensitivity and specificity.

Calibration of the base model and the new model incorporating LASr-c was evaluated using calibration slopes, intercepts, Brier scores, and calibration plots. For the new model, the bias-corrected calibration slope was 1.029 (95% CI: 0.791–1.331) and the intercept was 0.031 (95% CI: −0.469–0.642). The Brier score was 0.071 (95% CI: 0.052–0.089), reflecting good overall predictive accuracy. The corresponding calibration plot is provided in Supplementary Figure 2.

DCA (Figure 5) was performed with the clinical intervention defined as initiation of targeted prolonged cardiac monitoring for PAF screening. Results showed that the new model with LASr-c yielded higher net clinical benefit than the base model across most risk threshold ranges, confirming that incorporation of LASr-c can improve the clinical utility of noninvasive PAF screening in AIS patients.

**Figure 5:**
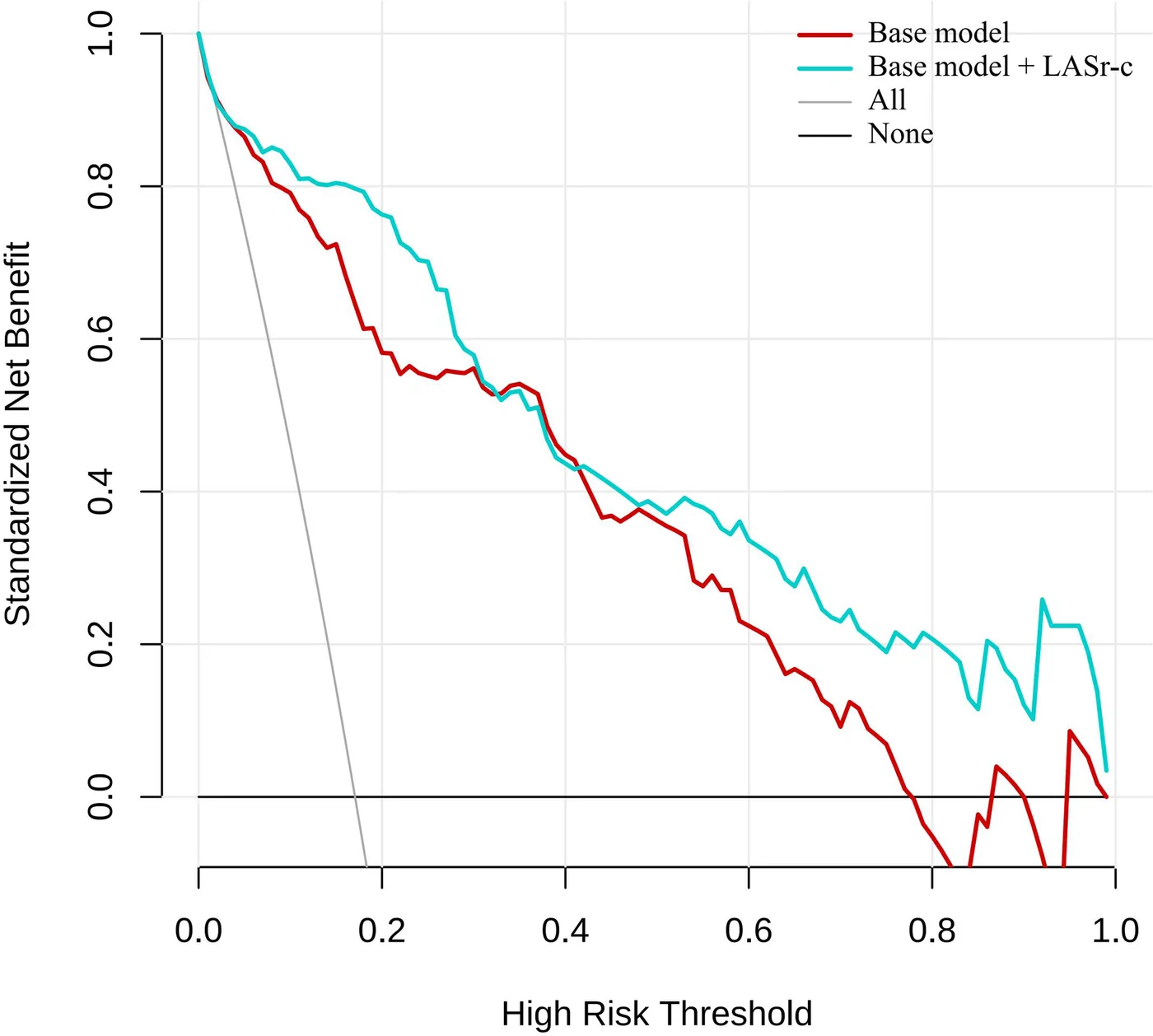
DCA comparing the clinical net benefit of the base diagnostic model and the model incorporating LASr-c. LASr-c, LA circumferential strain reservoir phase.

### 3.6 Sensitivity analyses

Consistent results across the both sensitivity analyses further support the robustness of our findings (Table 3). The diagnostic and incremental value of LASr-c persisted after excluding overt cardioembolic cases or after adjusting for more potential confounders.

**Table 3:** Sensitivity analyses.

Analysis scenario	Metric	Primary analysis	Sensitivity 1	Sensitivity 2
LASr-c	AUC (95% CI)	0.845 (0.780–0.911)	0.801 (0.701–0.901)	-
Base model	C-index	0.910 (0.867–0.953)	0.920 (0.876–0.963)	0.912 (0.869–0.955)
Base model + LASr-c	C-index	0.932 (0.893–0.971)	0.939 (0.906–0.971)	0.936 (0.897–0.974)
Incremental value	Continuous NRI (95% CI)	0.864 (0.622–1.105)	0.783 (0.433–1.132)	0.864 (0.622–1.105)
	IDI (95% CI)	0.097 (0.047–0.147)	0.082 (0.016–0.149)	0.098 (0.050–0.146)

Sensitivity 1: Excluding CE-TOAST cases (*n* = 296).

Sensitivity analysis 2: Extended covariate adjustment in the full cohort (*n* = 340), with adjustment for age, logBNP, LAVI, LVEF, E/e', serum creatinine, coronary heart disease, and beta-blocker use.

LASr-c, left atrial circumferential strain reservoir phase; AUC, area under the curve; CI, confidence interval; NRI, net reclassification improvement; IDI, integrated discrimination improvement.

## 4 Discussion

This study systematically investigated the diagnostic value of left atrial 3D strain parameters for PAF in hospitalized patients with early-stage AIS. The results showed that all left atrial 3D strain parameters exhibited good discriminatory performance for PAF, with circumferential strain during the reservoir and contractile phases showing the best diagnostic value. For the first time, we demonstrated that adding LASr-c to a clinical base model incorporating age, sex, logBNP, and LAVI can improve diagnostic performance, particularly risk reclassification ability and net clinical benefit, thus providing a promising new tool for noninvasive PAF screening in AIS patients.

AF is closely linked to LA structural and functional remodeling. Structural remodeling is primarily characterized by LA enlargement and myocardial fibrosis, whereas functional remodeling manifests as impairment of LA reservoir, conduit, and contractile functions (23). Previous studies predominantly used structural parameters such as LAVI and LA diameter to assess LA remodeling. However, in patients with PAF, especially those with concealed PAF, LA enlargement often occurs at a relatively late stage, while LA functional impairment can appear early in the course of AF (24). Histological studies by Lisi et al. have confirmed that even mild atrial fibrosis leads to a significant reduction in LA strain, even when LA volume remains within normal limits (25). Our results demonstrated that all left atrial 3D strain parameters were significantly lower in the PAF group than in the non-PAF group, suggesting that 3D strain technology is more sensitive in detecting early LA dysfunction in AIS patients with PAF. Furthermore, Soulat-Dufour et al. showed that LA strain serves as a memory marker of atrial myocardial stress induced by AF and directly reflects the severity of atrial fibrosis; LA functional impairment persists even after termination of the arrhythmia and restoration of sinus rhythm (11). In our study, echocardiography was performed at a median of 6 days after symptom onset, mostly concurrent with the 24-hour Holter recording. Most PAF episodes were detected during the full monitoring period including Holter. We cannot retrospectively quantify the exact number of patients with documented AF before echocardiography, as many episodes were asymptomatic and identified only by continuous rhythm review. However, the chronic nature of LA strain remodeling may support its role as a predictive risk marker, not just a post-AF imaging phenotype.

LA myocardial fibers are classified into three orientations: circumferential, longitudinal, and oblique. Circumferential fibers are predominantly located in the middle layer and are the core structures responsible for maintaining LA wall tension and ensuring diastolic reservoir function (26). Reservoir-phase LA circumferential strain accurately reflects the expansion capacity of circumferential fibers during left ventricular systole, directly capturing LA diastolic reserve (27). Myocardial fibrosis and impaired diastolic function are the fundamental pathological substrates for AF initiation and maintenance (7). Bao et al. reported that both LA longitudinal strain and LA circumferential strain can differentiate patients with paroxysmal AF from healthy (28). A likely explanation is that longitudinal strain only reflects the function of subendocardial longitudinal fibers and is susceptible to interference from left ventricular function and loading conditions, whereas circumferential strain reflects the function of the predominant circumferential fibers and is more sensitive to early atrial fibrosis (29). In our study, reservoir-phase LA circumferential strain yielded an AUC of 0.845 for diagnosing PAF, significantly outperforming longitudinal strain parameters, consistent with previous findings.

The base diagnostic model constructed in this study included age, sex, logBNP, and LAVI, achieving a C-index of 0.910, which already represents excellent discriminatory ability. After adding LASr-c, the C-index increased to 0.932. Although the DeLong test did not show a statistically significant difference, this likely stems from the fact that the base model had already approached a performance ceiling, substantially compressing the margin for AUC improvement and making it difficult to detect small yet clinically meaningful gains using AUC alone (30). Statistically, C-index/AUC reflects the overall discriminative ability of the model across the entire risk spectrum. In contrast, continuous NRI and IDI are metrics specifically designed to quantify the incremental value of a new biomarker, by directly measuring the net proportion of patients correctly reclassified. They are substantially more sensitive than AUC for detecting marginal improvements in risk prediction, especially when baseline performance is already high (31).

Clinically, for the scenario of PAF screening after AIS, the core goal is not simply to maximize global AUC, but to achieve more accurate risk stratification to guide decisions about prolonged rhythm monitoring. A significant positive NRI indicates that adding LASr-c produces a net improvement in individual risk classification: more patients with true PAF are reclassified into higher risk categories (reducing missed cases), while more patients without PAF are reclassified into lower risk categories (reducing unnecessary testing). This reclassification benefit is of greater practical relevance to clinical decision-making than a small increment in global AUC. A missed PAF diagnosis may delay the initiation of guideline-directed anticoagulation, leading to an increased risk of recurrent stroke, whereas false-positive results necessitate unnecessary downstream examinations, which consumes healthcare resources and increases patient burden (32, 33). This interpretation is further supported by the DCA, which shows higher net clinical benefit of the LASr-c-augmented model across most clinically relevant risk thresholds. We have to admit that LASr-c is not a diagnostic tool for PAF on its own and cannot be used to justify initiating anticoagulation without ECG-documented evidence of AF. These findings provide diagnostic evidence supporting the potential use of LASr-c as a noninvasive risk stratification tool to select AIS patients for targeted prolonged cardiac monitoring, such as ≥14-day cardiac rhythm monitoring (34). However, the actual clinical yield and utility of a strain-guided monitoring strategy remain to be validated in prospective interventional studies.

This study has several limitations. First and most critically, the reference standard for PAF detection has inherent constraints. Our monitoring strategy—including admission 12-lead ECG, ≥48 h of in-hospital continuous monitoring, and at least one 24-hour Holter recording (totaling ≥72 h of monitoring)—falls short of the ≥14-day cardiac monitoring recommended by the 2024 ACC Expert Consensus. Misclassification of occult PAF into the non-PAF group may occur. Importantly, this misclassification would most likely represent a conservative bias. Pathophysiologically, it is plausible that PAF cases missed by short-term monitoring have lower arrhythmia burden and less advanced atrial structural and functional remodeling, such that their LA strain values are closer to those of patients without PAF. If this is the case, inclusion of these patients in the non-PAF group would systematically attenuate true between-group differences and conservatively underestimate the model's discriminative capacity, reclassification performance, and net clinical benefit, rather than inflating our results. Nevertheless, we acknowledge that the actual remodeling status of these undetected cases remains unknown. We also acknowledge that the LASr-c cutoff value derived in this cohort is established based on short-term in-hospital detected PAF, and cannot be directly extrapolated to populations with PAF confirmed by extended ≥14-day monitoring or implantable loop recorders. Second, this single-center study with a limited sample size may introduce selection bias. Third, no external validation was performed, and the generalizability of the proposed model requires further verification. Finally, PAF episode burden was not systematically quantified, precluding assessment of its association with left atrial 3D strain. Large multicenter prospective studies with prolonged rhythm monitoring and external validation are warranted to confirm the true diagnostic performance and optimal cutoff of LASr-c for PAF detection.

## 5 Conclusion

Left atrial 3D strain demonstrates good diagnostic value for short-term in-hospital detected PAF in patients with AIS. Adding LASr-c to the base diagnostic model constructed from conventional clinical indicators can improve the model's diagnostic performance, particularly risk reclassification ability and net clinical benefit. Incorporation of LASr-c may improve PAF risk stratification among hospitalized AIS patients and provides a diagnostic basis for guiding subsequent targeted rhythm monitoring strategies, which require further prospective validation.

## Data Availability

The raw data supporting the conclusions of this article will be made available by the authors, without undue reservation.
