# Supplementary material for: Diagnostic value of left atrial three-dimensional strain for paroxysmal atrial fibrillation in patients with acute ischemic stroke
Source: Front Cardiovasc Med. 2026 Sep 23;13:1909840. doi: 10.3389/fcvm.2026.1909840 (PMC13642124; doi:10.3389/fcvm.2026.1909840)
Supplement: Supplementary file 1 [file Supplementaryfile1.docx]

Supplementary Table 1 Univariable ROC Curve Analysis

| Predictor | LASr, % | LAScd, % | LASct, % | LASr-c, % | LAScd-c, % | LASct-c, % |
| --- | --- | --- | --- | --- | --- | --- |
| AUC | 0.806 | 0.622 | 0.77 | 0.845 | 0.629 | 0.851 |
| 95% CI | 0.738 - 0.875 | 0.538 - 0.706 | 0.701 - 0.840 | 0.780 - 0.911 | 0.544 - 0.714 | 0.797 - 0.906 |
| Cutpoint | 16 | -7 | -7 | 20 | -4 | -11 |
| Youden | 0.527 | 0.254 | 0.456 | 0.603 | 0.245 | 0.622 |
| Sensitivity(%) | 82.8 | 53.4 | 79.3 | 75.9 | 36.2 | 84.5 |
| Specificity(%) | 69.9 | 72 | 66.3 | 84.4 | 88.3 | 77.7 |
| PPV(%) | 36.1 | 28.2 | 32.6 | 50 | 38.9 | 43.8 |
| NPV(%) | 95.2 | 88.3 | 94 | 94.4 | 87.1 | 96.1 |

Sign convention: Reservoir-phase strains (LASr, LASr-c) are positive (left atrial expansion); conduit- and contractile- phase strains (LAScd, LASct, LAScd-c, LASct-c) are negative (left atrial emptying). For all parameters, smaller absolute magnitude indicates impaired LA function and higher PAF risk.

LA: Left atrial; LASr: LA longitudinal strain reservoir phase; LAScd: LA longitudinal strain conduit phase; LASct: LA longitudinal strain contraction phase; LASr-c: LA circumferential strain reservoir phase; LAScd-c: LA circumferential strain conduit phase; LASct-c: LA circumferential strain contraction phase; PPV: Positive predictive value; NPV: Negative predictive value.

Supplementary Table 2 Univariable Logistic Regression

| **Characteristic** | **OR** | **95% CI** | **p-value** |
| --- | --- | --- | --- |
| Age, years | 1.05 | 1.02, 1.08 | <0.001 |
| Sex, male, n (%) | 0.66 | 0.37, 1.18 | 0.158 |
| BMI, kg/m² | 1.02 | 0.95, 1.10 | 0.564 |
| Smoking, n (%) | 0.53 | 0.27, 1.04 | 0.064 |
| Alcohol consumption, n (%) | 0.48 | 0.18, 1.28 | 0.142 |
| Hypertension, n (%) | 1.6 | 0.82, 3.11 | 0.169 |
| Diabetes mellitus, n (%) | 0.65 | 0.34, 1.24 | 0.189 |
| Coronary heart disease, n (%) | 3.06 | 1.22, 7.68 | 0.017 |
| Prior stroke, n (%) | 1.31 | 0.67, 2.57 | 0.425 |
| Antiplatelet agents, n (%) | 1.63 | 0.88, 3.01 | 0.121 |
| Statin, n (%) | 1.35 | 0.74, 2.47 | 0.325 |
| ACEI/ARB, n (%) | 1.24 | 0.61, 2.51 | 0.55 |
| β-blockers, n (%) | 2.91 | 1.32, 6.43 | 0.008 |
| Time from symptom onset to admission, hours | 0.99 | 0.98, 1.00 | 0.03 |
| Time from admission to Holter, days | 0.99 | 0.90, 1.08 | 0.779 |
| Infarct location, n (%) |  |  |  |
| Cortical | — | — |  |
| Deep | 0.3 | 0.15, 0.57 | <0.001 |
| Multiple | 0.46 | 0.17, 1.23 | 0.121 |
| Negative | 0.84 | 0.33, 2.12 | 0.712 |
| Infarct size, cm^2^ | 1.2 | 0.93, 1.54 | 0.166 |
| TOAST classification, n (%) |  |  |  |
| LAA | — | — |  |
| CE | 20.62 | 9.09, 46.79 | <0.001 |
| SAA | 0.83 | 0.35, 1.93 | 0.663 |
| SOE | 0 | 0.00, Inf | 0.988 |
| SUE | 0.67 | 0.08, 5.40 | 0.703 |
| OCSP classification, n (%) |  |  |  |
| TACI | — | — |  |
| PACI | 0.88 | 0.31, 2.44 | 0.799 |
| POCI | 0.49 | 0.16, 1.49 | 0.211 |
| LACI | 0.71 | 0.25, 1.97 | 0.508 |
| Admission NIHSS score | 1.06 | 0.99, 1.13 | 0.115 |
| White blood cell count, ×10⁹/L | 1.08 | 0.96, 1.22 | 0.216 |
| Red blood cell count, ×10¹²/L | 0.64 | 0.39, 1.07 | 0.091 |
| Hemoglobin, g/L | 0.98 | 0.97, 1.00 | 0.054 |
| Platelet count, ×10⁹/L | 1 | 0.99, 1.00 | 0.538 |
| Fibrinogen, g/L | 1.34 | 0.98, 1.82 | 0.065 |
| D-dimer, mg/L | 1.12 | 0.93, 1.36 | 0.221 |
| CK-MB, U/L | 1.01 | 1.00, 1.03 | 0.117 |
| Myoglobin, ng/mL | 1 | 0.99, 1.00 | 0.112 |
| Troponin I, ng/L | 1.05 | 1.02, 1.08 | 0.001 |
| BNP, pg/mL | 1 | 1.00, 1.00 | <0.001 |
| CRP, mg/L | 1.01 | 0.99, 1.02 | 0.603 |
| Serum creatinine, μmol/L | 1 | 1.00, 1.00 | 0.882 |
| Glucose, mmol/L | 0.96 | 0.86, 1.09 | 0.543 |
| Total cholesterol, mmol/L | 0.69 | 0.52, 0.91 | 0.01 |
| Triglycerides, mmol/L | 1.01 | 0.99, 1.03 | 0.268 |
| HDL-C, mmol/L | 0.8 | 0.42, 1.53 | 0.496 |
| LDL-C, mmol/L | 0.81 | 0.61, 1.05 | 0.116 |
| Homocysteine, μmol/L | 1.01 | 0.99, 1.04 | 0.325 |
| HbA1c, % | 0.97 | 0.84, 1.12 | 0.648 |
| Time from symptom onset to LA strain measurement, days | 0.98 | 0.89, 1.08 | 0.658 |
| Heart rate, beat per minutes | 1.02 | 0.99, 1.04 | 0.166 |
| LAD, mm | 1.21 | 1.14, 1.29 | <0.001 |
| LVEDD, mm | 1.1 | 1.03, 1.17 | 0.003 |
| LVEF, % | 0.94 | 0.90, 0.98 | 0.003 |
| IVST, mm | 0.98 | 0.79, 1.21 | 0.825 |
| LVPWT, mm | 1 | 0.77, 1.30 | 0.979 |
| E/e' ratio | 1.07 | 0.98, 1.17 | 0.147 |
| LAVI, mL/m² | 1.19 | 1.13, 1.24 | <0.001 |
| LASr, % | 0.84 | 0.80, 0.89 | <0.001 |
| LAScd, % | 1.06 | 1.01, 1.12 | 0.031 |
| LASct, % | 1.18 | 1.12, 1.25 | <0.001 |
| LASr-c, % | 0.87 | 0.84, 0.90 | <0.001 |
| LAScd-c, % | 1.04 | 1.00, 1.09 | 0.044 |
| LASct-c, % | 1.17 | 1.12, 1.22 | <0.001 |

BMI: Body mass index; ACEI: Angiotensin-converting enzyme inhibitor; ARB: Angiotensin receptor blocker; TOAST: Trial of Org 10172 in Acute Stroke Treatment; LAA: Large-artery atherosclerosis; CE: Cardioembolism; SAA: Small-artery occlusion; SOE: Stroke of other determined etiology; SUE: Stroke of undetermined etiology; OCSP: Oxfordshire Community Stroke Project; TACI: Total anterior circulation infarct; PACI: Partial anterior circulation infarct; POCI: Posterior circulation infarct; LACI: Lacunar infarct; NIHSS: National Institutes of Health Stroke Scale; CK-MB: Creatine kinase-MB; BNP: B-type natriuretic peptide; CRP: C-reactive protein; HDL-C: High-density lipoprotein cholesterol; LDL-C: Low-density lipoprotein cholesterol; HbA1c: Hemoglobin A1c; LA: Left atrial; LAD: LA diameter; LVEDD: Left ventricular end-diastolic diameter; LVEF: Left ventricular ejection fraction; IVST: Interventricular septal thickness; LVPWT: Left ventricular posterior wall thickness; LAVI: LA maximum volume index; LASr: LA longitudinal strain reservoir phase; LAScd: LA longitudinal strain conduit phase; LASct: LA longitudinal strain contraction phase; LASr-c: LA circumferential strain reservoir phase; LAScd-c: LA circumferential strain conduit phase; LASct-c: LA circumferential strain contraction phase.

Supplementary Table 3 Multivariable Logistic Regression

| **Characteristic** | **OR** | **95% CI** | **p-value** |
| --- | --- | --- | --- |
| Basic model | | | |
| Age, years | 1.02 | 0.98, 1.06 | 0.286 |
| Sex, male, n (%) | 1.23 | 0.56, 2.71 | 0.608 |
| logBNP, pg/mL | 5.65 | 2.57, 12.39 | <0.001 |
| LAVI, mL/m² | 1.16 | 1.11, 1.22 | <0.001 |
| Basic model + LASr-c | | | |
| Age, years | 1.00 | 0.97, 1.04 | 0.846 |
| Sex, male, n (%) | 1.44 | 0.59, 3.52 | 0.42 |
| logBNP, pg/mL | 1.74 | 0.72, 4.19 | 0.22 |
| LAVI, mL/m² | 1.18 | 1.12, 1.25 | <0.001 |
| LASr-c, % | 0.88 | 0.84, 0.93 | <0.001 |

BNP: B-type natriuretic peptide; LA: Left atrial; LAVI: LA maximum volume index; LASr-c: LA circumferential strain reservoir phase.

Notes:

Predicted-risk equation derived from multivariable logistic regression:

**Basic model:** log[p/(1-p)] = -12.234 + 0.019×Age + 0.207×Sex(male: 1; female:0) + 1.731×logBNP + 0.148×LAVI
**Basic model +LASr-c:** log[p/(1-p)] = -6.449 + 0.004×Age + 0.367×Sex(male: 1; female:0) + 0.551×logBNP + 0.167×LAVI - 0.127×LASr-c

**Supplementary Figure 1 Intra-observer and inter-observer agreement assessment**
The upper panel displays Bland-Altman plots for inter-observer agreement assessment, with corresponding intraclass correlation coefficient (ICC) values as follows: LASr: 0.962; LAScd: 0.969; LASct: 0.942; LASr-c: 0.978; LAScd-c: 0.895; LASct-c: 0.880.

The lower panel displays Bland-Altman plots for intra-observer agreement assessment, with corresponding ICC values as follows: LASr: 0.946; LAScd: 0.923; LASct: 0.930; LASr-c: 0.972; LAScd-c: 0.823; LASct-c: 0.933.

**Supplementary Figure 2 Calibration curves for the base model and the base model + LASr-c**

The left panel shows the calibration plot of the **base model** based on 1000 bootstrap resamples, with a calibration slope of 1.026 (95% CI: 0.736-1.348), an intercept of 0.022 (95% CI: −0.481 to 0.574), and a Brier score of 0.082 (95% CI: 0.061-0.103).

The right panel shows the calibration plot of the **base model+LASr-c** based on 1000 bootstrap resamples, with a calibration slope of 1.029 (95% CI: 0.791-1.331), an intercept of 0.031 (95% CI: −0.469 to 0.642), and a Brier score of 0.071 (95% CI: 0.052-0.089).
